# Anti-Inflammatory and Antinociceptive Properties of Flavonoids from the Fruits of Black Mulberry (*Morus nigra* L.)

**DOI:** 10.1371/journal.pone.0153080

**Published:** 2016-04-05

**Authors:** Hu Chen, Junsong Pu, Dan Liu, Wansha Yu, Yunying Shao, Guangwei Yang, Zhonghuai Xiang, Ningjia He

**Affiliations:** State Key Laboratory of Silkworm Genome Biology, Southwest University, Chongqing, 400715, China; Temple University, UNITED STATES

## Abstract

We analyzed the anti-inflammatory and antinociceptive activities of total flavonoids (TF) found in black mulberry fruits. The TF content was 20.9 mg/g (dry weight). Two anthocyanins, cyanidin-3-O-glucoside (8.3 mg/g) and cyanidin-3-O-rutinoside (2.9 mg/g), were identified in the fruits by UPLC. The TF of black mulberry fruits had significant reducing power and radical (OH^-^,${\text{ O}}_{2}^{.-}$, DPPH and ABTS) scavenging activities that was demonstrated in a dose-response curve. The TF had inhibitory activities on xylene-induced ear edema and carrageenan-induced paw edema in mice. In addition, TF had antinociceptive activities in the two nociceptive phases of formalin test. We used ELISA to detect the pro-inflammatory cytokines IL-1β, TNF-α, IFN-γ, and NO in the serum of mice. These cytokines were significantly inhibited or scavenged by TF (50 and 100 mg/kg). The results demonstrated that TF of black mulberry possess anti-inflammatory and analgesic effects that might correlate to its antioxidant activities and inhibition of pro-inflammatory cytokines.

## Introduction

Black mulberry, *M*. *nigra*, is the *Morus* species with the highest chromosome number [1]. It originated in Iran, but is grown throughout southern Europe, southwest Asia, the Mediterranean countries, and South America [2,3]. This species is the only black mulberry cultivated in China and is found mainly in the Aksu, Hotan, and Kashi districts in Xinjiang Uygur Autonomous Region. It is named Xiatutu in the Uygur region, which means medicinal mulberry.

Uighurs like eating meat, milk, and pop barbecue, but consume few vegetables. Therefore they are especially prone to respiratory and digestive diseases. Medicine mulberry is an Uygur folk medicine and is used to treat tonsillitis and sore throat [4]. Uighurs still retain the tradition of making medicinal mulberry cream that can be used year round. Many studies have found that black mulberry is rich in polyphenols, flavonoids, and anthocyanins. The contents of anthocyanins in medicinal mulberry are significantly higher than those in other varieties of mulberry such as red and white [4–6]. Black mulberry has antioxidant, anti-cancer, and hypoglycemic activities with non-toxic side effects [7,8].

Inflammation is an immunological defense mechanism that the body uses to fight bacteria, viruses, and other pathogens [9,10]. In these processes, a variety of chemical mediators are released from damaged tissue including excitatory amino acids, hydrogen ions, peptides, lipids, and cytokines. These underlie inflammation and pain [11]. Pro-inflammatory cytokines will continue to damage tissue if they are not cleared from the body. Redness, warmth, swelling and pain are the classic clinical features of inflammation [12]. Non-steroidal anti-inflammatory drugs (NSAIDs) such as aspirin and steroidal anti-inflammatory drugs (SAIDs) such as dexamethasone have been widely used to combat inflammation, but also suffer significant side effects for instance gastrointestinal disorders [9,13]. As an alternative, natural medicines are getting increasing attention due to their mild action and lower side effects.

Ma (2002) reported that the fruits of the medicinal mulberry were used to treat pharyngitis patients [14]. Another study proved that mulberry anthocyanins had good anti-inflammatory effects [15]. Despite those traditional claims regarding the medicinal mulberry, the scientific evidences supporting these pharmacological and phytochemical claims has not been clearly demonstrated. The aim of this study is to investigate the anti-inflammatory and antinociceptive activities of total flavonoids from the fruits of medicinal mulberry in animal models.

## Materials and Methods

### Mulberry fruits

The fruits of the black mulberry (*M*. *nigra*) were collected from the mulberry germplasm nursery in Hetian Sericultural Research Institute (N37°08'50.85" E79°54'26.99") of Xinjiang Uygur Autonomous Region, China. The fruits were oven-dried at 60°C to a constant mass and then pulverized. The powders were sieved to pass through a 60 mesh sieve and stored at -40°C until used.

### Animals

Kunming (KM) male mice weighing 18–22 g were purchased from Chongqing Medical University, China. The license number of experimental animals is SCXK (YU) 2012–0006. The mice were maintained in our animal facilities under standard conditions (21±2°C, 12 h light/dark cycle) with a pelleted mouse diet (HFK Bioscience, Beijing) and reverse osmosis water *ad libitum*. All animal treatments were in strict accordance with international ethical guidelines concerning the care and use of laboratory animals. The Animal Care Committee of Southwest University approved this study.

### Chemicals and reagents

Aspirin (Asp) was purchased from Yabao (Shanxi, China) and dexamethasone (Dex) was obtained from Xianju Pharma (Zhejiang, China). Anthocyanin standard cyanidin-3-O-glucoside (C3G), cyanidin-3-O-rutinoside (C3R) and lipopolysaccharide (LPS) were purchased from Sigma-Aldrich (St. Louis, MO, USA). Flavonoid standard quercetin-3-O-rutinlside (rutin) was obtained from the National Institutes for Food and Drug Control (Beijing, China). Acetonitrile (ACN) and methanol (MeOH) for UPLC analysis were purchased from Thermo Fisher Scientific (Waltham, MA, USA). Other analytical grade chemicals were obtained from the Chengdu Kelong Chemical Reagent Factory (Sichuan, China). High performance liquid chromatography (HPLC) grade water was from a Milli-Q System (Millipore, Billerica, MA, USA) and 0.22 μm Millipore membranes were purchased from Sangon Biotech (Shanghai, China).

The enzyme-linked immunosorbent assay (ELISA) kits used to determine mouse interleukin 1β (IL-1β), tumor necrosis factor α (TNF-α), interferon γ (IFN-γ) and nitric oxide (NO) were purchased from CUSABIO (Wuhan, China). The nitric oxide test kit in cell culture was purchased from Nanjing Jiancheng Bioengineering Institute (Nanjing, China). Fetal bovine serum (FBS), Dulbecco’s modified eagle medium (DMEM), antibiotics (streptomycin/penicillin), and trypsin were purchased from Gibco (Grand Island, NY, USA). The 3-(4,5-dimethylthiazol-2-yl)-2,5-diphenyltetrazolium bromide (MTT) and Griess reagent were purchased from Beyotime (Shanghai, China).

### Extraction of total flavonoids

The fruit powder (100 g) was extracted with petroleum ether (1,000 mL, boiling range from 30–60°C) using a Soxhlet apparatus at 70°C for 6 h. The skim powder was dried in a vacuum desiccator for 24 h to remove the petroleum ether. This was followed by an ultrasonic extraction with 38% (v:v) ethanol for 15 min at 60°C [16]. The liquid was separated from the solid matrix by centrifugation (4000 g) at 4°C for 15 min. The supernatant was collected, and the solid matrix was re-extracted once. The resulting supernatant was combined and concentrated to a final volume of 200 mL in a rotary evaporator. The extraction of total flavonoids, referred to as TF, was stored at -20°C until further use.

### Concentration determination of total flavonoids (TF)

TF concentration was measured using the method reported by Chen et al. [16]. Briefly, 0.6 mL of TF, 4.4 mL of 60% (v/v) ethanol solution, and 0.3 mL of 5% (w/v) NaNO_2_ solution were mixed in a 10 mL volumetric flask. After 6 min of shaking, 0.3 ml of 10% (w/v) Al(NO_3_)_3_ solution was added and shaken for another 6 min. 4 mL of 4% (w/v) NaOH solution was then added and 60% (v/v) ethanol solution was added to a final volume of 10 ml. The absorption at 510 nm was measured using a Techcomp UV1000 spectrophotometer (Shanghai, China). A standard curve was generated in parallel with a rutin standard. TF concentration was calculated as mg/mL of rutin equivalents.

### Quantification of anthocyanins and rutin by UPLC

Standards of C3G, C3R and rutin were accurately weighed and dissolved in ACN/H_2_O/HCL (1:7:2, v:v:v; HCL, 0.1 mol/L), ACN/HCL (2:98, v:v; HCL, 0.01 mol/L), and H_3_PO_4_/MeOH (1:999, v:v), respectively. Standard solutions were diluted to the appropriate concentrations and ranged from 1.56 to 25 μg/mL for C3G and C3R, and 6.25 to 100 μg/mL for rutin. The chromatographic separation was carried out on a Waters Acquity UPLC I-Class system including a TUV detector, a sample manager-FTN, and an Acquity UPLC BEH C18 column (2.1×100 mm, 1.7 μm, Waters, Milford, MA).

We used a binary mobile phase as the rutin solvent. Solution A was MeOH and solution B was Milli-Q water containing 0.1% (v:v) H_3_PO_4_. The linear elution gradient was as follows: 0–2.73 min, 30% A, curve 1; 2.73–9.4 min, 30–40% A, curve 6; 9.4–11.4 min, 40–50% A, curve 6; and 11.4–15 min, 50–30% A, curve 1. These were all at a flow rate of 0.21 mL/min. The column temperature was kept at 30°C. The injection volume was 4 μL, and the detection wavelength was 358 nm. To measure anthocyanins, a binary mobile phase was also used. Here, solution A was ACN and solution B was Milli-Q water containing 0.2% (v:v) H_3_PO_4_. The linear elution gradient was as follows: 0–8 min, 7–13% A, Curve 6; 8–9 min, 13–7% A, curve 1. This was at a flow rate of 0.3 mL/min. The column temperature was kept at 30°C. The injection volume was 1.4 μL. The detection wavelength was 520 nm.

### Antioxidant activity

In the following antioxidant tests, samples of black mulberry extract were diluted with 38% ethanol, to obtain a TF concentration of 0.048 mg/mL. The concentration of Vc (control) was 0.048 mg/mL.

#### Determination of reducing power

The reducing power was determined according to a known protocol with minor modifications [17]. Different volumes (0.05, 0.1, 0.2, 0.3, 0.4 mL) of the sample and Vc were respectively added to a brown 10 mL volumetric flask and then mixed with 1 mL of 10 mg/mL potassium ferricyanide and phosphate buffer (pH 6.5). The mixture was incubated at 50°C for 20 min. After 1 mL of trichloroacetic acid (100 mg/mL) was added, the sample and Vc were allowed to incubate for 10 min. Then 1 mL of ferric chloride (1 mg/mL) and 1 mL of distilled water were added and constant volume ethanol, the solution was reacted for another 10 min. The absorbance was measured at 700 nm. The reducing power was calculated based on the absorbance.

#### Determination of hydroxyl radical (OH-) scavenging activity

The OH^-^ scavenging activity was determined by a method provided by Chen et al. [16]. The mixture, with 2 mL of 2 mM FeSO_4_, 2 mL of 1 mM H_2_O_2_ and 3 mL of 6 mM salicylic acid were added to a 10 mL centrifuge tube and incubated at 37°C for 15 min. The absorbance of the mixture was measured at 510 nm. One mL of concentration gradient (9.6, 19.2, 28.8, 38.4, 48μg/mL) of the sample and Vc were then respectively added to the mixture and incubated at 37°C for 15 min. The absorbance was measured again at 510 nm. For the control, the H_2_O_2_ solution was replaced with distilled water. The hydroxyl radical scavenging rate was calculated as follows:

$$\text{Scavenging activity}/\%=\frac{A_{\text{OH}}^{-}-\left(A_{\text{s}}-A_{\text{s}0}\right)}{A_{\text{OH}}^{-}}\times 100$$

Here,$A_{\text{OH}}^{-}$ is the absorbance of the mixture without sample; *A*_S_ is the absorbance of the mixture with sample; and *A*_s0_ is the absorbance of sample without H_2_O_2_.

#### Determination of superoxide anion radical (${\text{ O}}_{2}^{.-}$) scavenging activity

The **${\text{ O}}_{2}^{.-}$** radical scavenging activity was determined with method reported by Roubaud ea al. with slight modifications [18]. The concentration gradient of sample and Vc were the same above (OH- test). The mixture, sample or Vc and 4.5 mL of 50 mM Tris-HCl buffer (pH 8.2), was added to a 10 mL centrifuge tube. The mixture was shaken and incubated at 25°C for 20 min. Preheated (25°C) 0.3 mL of 3 mM pyrogallol solution was then added. After standing for 5 min, 0.2 mL of 10 M of HCl was added to stop the reaction. The absorbance was measured at 325 nm. As a control, 0.3 mL of 10 mM HCl replaced the pyrogallol solution. The superoxide anion radical scavenging rate was calculated as follows:

$$\text{Scavenging activity/\%}=\frac{(A_{{\text{O}}_{2}}^{-}-A_{0})-\left(A_{\text{s}}-A_{\text{s}0}\right)}{A_{{\text{O}}_{2}}^{-}-A_{0}}\times 100$$

Here, $A_{{\text{O}}_{2}}^{-}$ is the absorbance of the mixture without sample; *A*_0_ is the absorbance of the mixture without sample and pyrogallol solution; *A*_S_ is the absorbance of the mixture with sample; and *A*_s0_ is the absorbance of the sample without pyrogallol.

#### Determination of 1, 1-diphenyl-2-picryl hydroxyl (DPPH) radical scavenging activity

DPPH radical scavenging activity was determined with a literature method with minor modifications [19,20]. Serial volumes of 2, 5, 10, 20, 30, 40, and 50 μL of samples and Vc were added into 96 well microtiter plates. Then, 20 μL of DPPH free radical agent (0.0158 g dissolved in 50 mL 95% (v:v) ethanol) and 95% (v:v) ethanol were added to a total volume of 200 μL and incubated at 37°C for 30 min. The absorbance was measured with a microplate reader at 540 nm. The DPPH radical scavenging ability was calculated as follows:

$$\text{Scavenging activity}/\%=\frac{A_{\text{DPPH}}-\left(A_{\text{s}}-A_{\text{s}0}\right)}{A_{\text{DPPH}}}\times 100$$

Here, *A*_DPPH_ is the absorbance of the DPPH solution with ethanol instead of sample; *A*_S_ is the absorbance of DPPH solution with sample; and *A*_S0_ is the absorbance of the sample with 95% (v:v) ethanol instead of DPPH solution.

#### Determination of 2, 2′-azinobis (3-ethylbenzothiazoline-6-sulfonic acid) (ABTS) radical cation antioxidant activity

The ABTS radical cation antioxidant activity was measured by a literature method with slight modifications [21]. The 2 mM ABTS and 2.45 mM potassium persulfate were mixed and incubated at 25°C for 4 h. The mixture was then diluted with phosphate buffer (0.1 M, pH 7.4) to an absorbance of 0.75 ± 0.025 nm as the working ABTS^+^ solution. Serial volumes of 2, 5, 10, 20, 30, 40, and 50 μL of samples and Vc were then added to the working ABTS^+^ solution, ethanol were added to a total volume of 150 μL and incubated at 25°C for 30 min. The absorbance was measured at 750 nm. The ABTS radical cation antioxidant activity was calculated as follows:

$$\text{Antioxidant capacity}/\text{\%}=\frac{A_{\text{ABTS}}^{.+}-\left(A_{\text{s}}-A_{\text{s}0}\right)}{A_{\text{ABTS}}^{.+}}\times 100$$

Here, $A_{\text{ABTS}}^{.+}$ is the absorbance of the ABTS^+^ solution with ethanol instead of sample; *A*_s_ is the absorbance of the ABTS^+^ solution with sample; and *A*_s0_ is the absorbance of the sample in phosphate buffer instead of ABTS^+^ solution.

### Evaluation of anti-inflammatory activity of black mulberry fruits

#### Xylene-induced ear edema in mice

The xylene-induced ear edema mouse model was conducted according to literature with minor modifications [22]. KM male mice were divided into six groups. Control: normal saline (20 mL/kg); Asp: aspirin (75 mg/20 mL/kg); Dex: dexamethasone (3 mg/20 mL/kg); TF-50 (50 mg/20 mL/kg); TF-100 (100 mg/20 mL/kg); and TF-200 (200 mg/20 mL/kg). The mice were maintained on adaptive feeding for 5 days prior to gavage. The vehicle and drugs were administered by gavage to the corresponding groups of mice once per day for 7 days. One hour after the last dose, the ventral and dorsal sides of the right ears were topically treated with 200 μL of xylene. One hour after xylene treatment, the mice were sacrificed and 6 mm punches were made in both ears. Each ear disc was weighed. The edema rate of the mice was calculated as follows: Edema rate (%) = [(1– Wa / Wb)] × 100, where Wb is the weight before xylene application and Wa is the weight after xylene application.

#### Carrageenan-induced paw edema in mice

Carrageenan-induced paw edema in mice was conducted according to the literature with minor modifications [11]. The grouping and gavage of mice was conducted using the same methods mentioned above (xylene-induced ear edema test). One hour after the last administration of drugs, the right paws of mice were topically injected subcutaneous by 10 μL of carrageenan. Four hours after carrageenan treatment, paw edema and the contralateral paw were measured with a Vernier caliper. The edema rate of mice was calculated as follows: Edema rate (%) = [(1– Hr / Hl)] × 100, where Hl is the height of left paw and Hr is the height of right paw.

### Evaluation of antinociceptive activity of black mulberry fruits

Formalin-induced pain-like behavior in mice was performed according to the literature [11,23]. The grouping and gavage of mice were conducted using the same methods mentioned in xylene-induced ear edema test. Ten microliters of 2.5% (v:v) formalin solution were injected subcutaneous into the left hind paw (n = 10 mice). After formalin injection, the treated mice were placed in separate boxes, and pain-like behaviors (licking, biting and flinching) were recorded in an initial phase (neurogenic phase, 0–5 min) and a secondary phase (inflammatory phase, 15–30 min).

### Immunological procedures

#### Blood collection

KM male mice were divided into six groups. Control: normal saline (20 mL/kg); Model: normal saline (20 mL/kg, injury group); Asp: aspirin (75 mg/20 mL/kg); Dex: dexamethasone (3 mg/20 mL/kg); TF-50 (50 mg/20 mL/kg); and TF-100 (100 mg/20 mL/kg). The mice were maintained on adaptive feeding for 5 days prior to gavage. Similar methods were used to administer vehicle and drugs. One hour after the last dose, the ventral and dorsal sides of the right ears in all groups except control group were topically treated by 200 μL of xylene as well as an intraperitoneal injection (i.p.) of 0.2 mL of 0.6% (v/v) acetic acid. Three hours later, blood was collected retroorbitally using serum separator tubes. Blood samples were clotted overnight at 4°C and then centrifuged for 15 min at 1,000 g to collect the serum. The serum was immediately assayed or stored at -40°C.

#### Cytokines and nitrite assays

The IL-1β, TNF-α, IFN-γ and NO serum measurement were performed with a two-site, sandwich enzyme-linked immunosorbent assay (ELISA) according to manufacturer’s instructions (CUSABIO, Wuhan, China). Cell culture nitrite was measured with a nitric oxide assay kit from Nanjing Jiancheng Bioengineering Institute (Nanjing, China).

### Cell culture, NO and cytotoxicity assay

The RAW 264.7 primary mouse macrophage cell line was purchased from Procell (Wuhan, China). The cells were cultured in DMEM with 10% (v:v) FBS and 1% (v:v) penicillin-streptomycin and incubated in a humidified atmosphere with 5% (v:v) CO_2_ at 37°C. NO and cytotoxicity were determined by the Griess method and MTT method according to manufacturer’s instructions, respectively.

### Statistical analyses

All data were expressed as the mean ± standard deviation (SD) and analyzed with SPSS statistical software (SPSS Inc., Chicago, IL, USA). A one-way ANOVA with Duncan’s test was used for inter-group comparison. The level of significance was set at p < 0.05 and p < 0.01.

## Results

### Determinations of TF, rutin and anthocyanins

**Fig 1:**
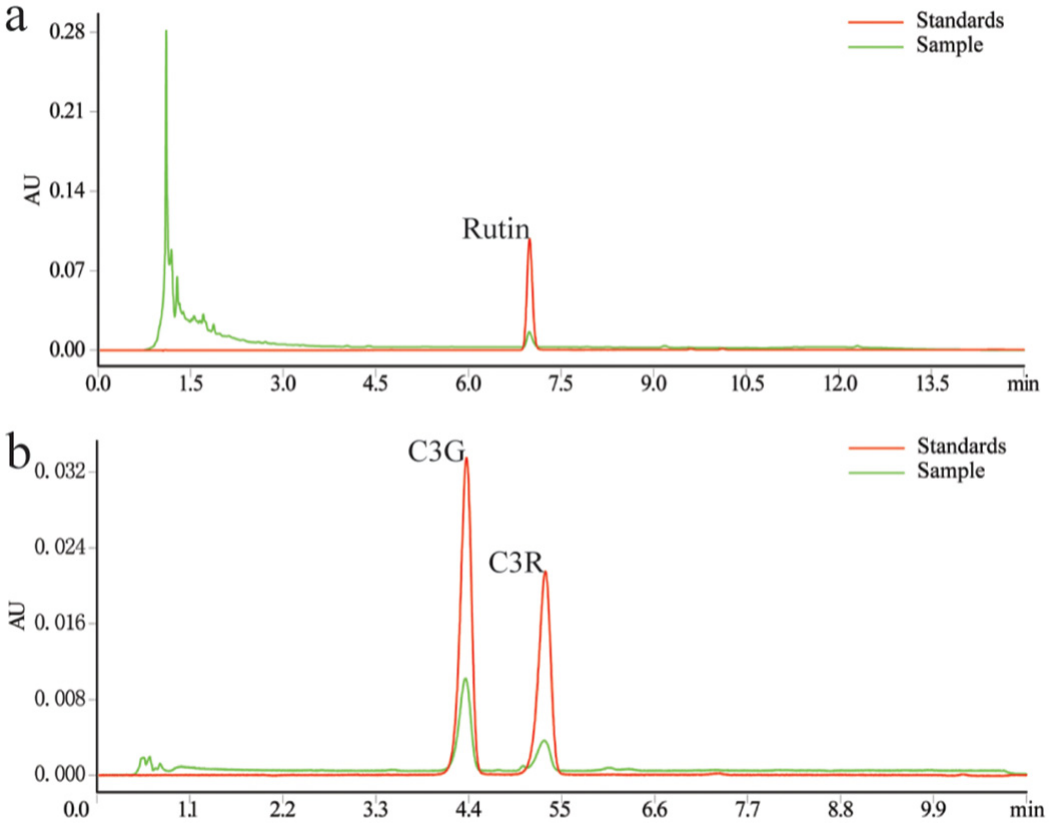
Chromatograms of rutin (A) and anthocyanins (B) of TF by UPLC-TUV. Rutin and anthocyanins were separated on the same Acquity UPLC BEH C18 column (2.1x100 mm, 1.7 μm, Waters, Milford, MA). Rutin separation used MeOH and 0.1% H_3_PO_4_ as the mobile phase at 0.21 mL/min with detection at 358 nm. Anthocyanins separation used ACN and 0.2% H_3_PO_4_ as the mobile phase at 0.3 mL/min and detection at 520 nm. Rutin: quercetin-3-O-rutinlside; C3G: cyanidin-3-O-glucoside; C3R: cyanidin-3-O-rutinoside; and TF: total flavonoids.

We measured the TF, rutin, and anthocyanin in the black mulberry fruits (Table 1). The TF was 20.9 mg/g. This was calculated as rutin equivalents and measured at 510 nm. Rutin was quantified using UPLC-TUV at 358 nm, C3G and C3R anthocyanin at 520 nm—mean level were 0.45 mg/g, 8.29 mg/g, and 2.89 mg/g, respectively. This result indicated that anthocyanins were the main ingredients of TF. We noted good linearity in the calibration curves of the 3 monomeric flavonoids (R^2^ > 0.9999). As shown in Fig 1a and 1b, our UPLC method completely resolved C3G and C3R with an effective separation of R = 3.657.

**Table 1:** Total flavonoids, anthocyanins and rutin content in the fruits of *Morus nigra* L. (n = 3).

Flavonoids	RT^a^ (min)	Regression equation^b^	R^2^	Content (mg/g)^c^
Total flavonoids	-	y = 10.635x + 0.0014	0.9995	20.8703 ± 0.9091^d^
Rutin	6.979	y = (6.065x + 2.3621)*10^3^	0.9999	0.4538 ± 0.0002^e^
Cyanidin-3-O-glucoside	4.408	y = (12.499x + 1.2386)*10^3^	0.9999	8.2941 ± 0.0014^e^
Cyanidin-3-O-rutinoside	5.345	y = (8.765x + 1.5500)*10^3^	0.9999	2.8896 ± 0.0009^e^

^*a*^ RT, Retention time.

^*b*^ y, Peak area; x, Concentration injected (μg/mL).

^*c*^ mg/g, weight in the dry powder of *Morus nigra* L.

^*d*^ The content calculated as rutin equivalents and measured by spectrophotometer.

^*e*^ The content measured by UPLC-TUV.

### Antioxidant activities

**Fig 2:**
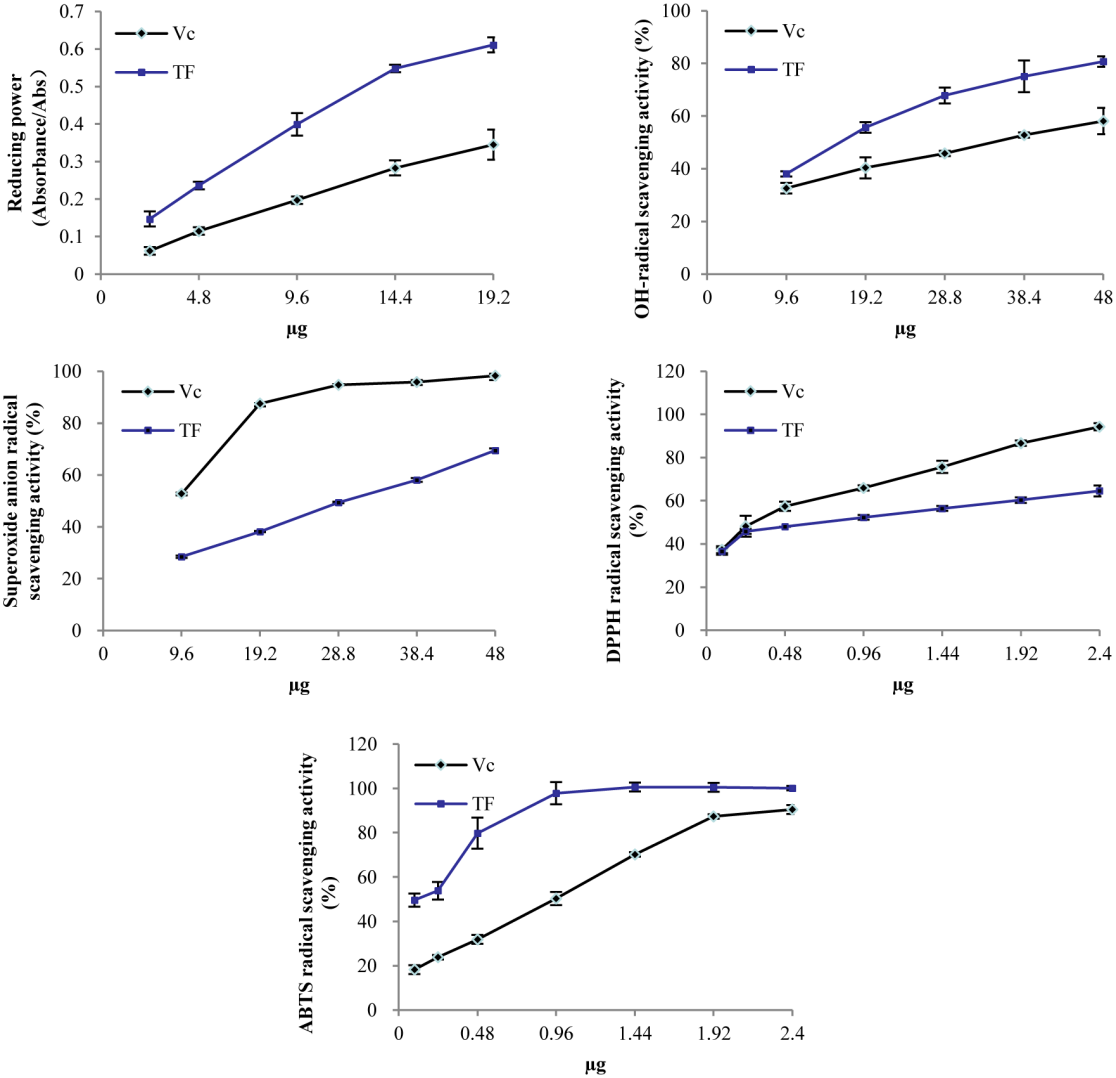
Antioxidant activities of TF. Data were mean ± SD (n = 3). We measured reducing power (a), OH- radical scavenging activity (b), ${\text{O}}_{2}^{.-}$ radical scavenging activity (c), DPPH radical scavenging activity (d), and ABTS radical scavenging activity (e).

We performed several *in vitro* antioxidant assays to measure the reducing power and radical (OH^-^,${\text{O}}_{2}^{.-}$, DPPH and ABTS) scavenging activities. This confirmed the antioxidant activity of TF. Versus vitamin C (Vc) (Fig 2a), TF had better reducing power with a dose-dependent pattern. The radical scavenging activities of OH^-^ (Fig 2b) and ABTS (Fig 2e) of TF were also comparably higher than that of Vc. The ABTS scavenging activity was very strong in both. At only 20 μL of TF, the scavenging rate for ABTS was 97.8%. The radical scavenging activities of TF and Vc were also measured across a range of ${\text{O}}_{2}^{.-}$ (Fig 2c) and DPPH (Fig 2d) concentrations, Vc was a better scavenger than TF.

### Anti-inflammatory and antinociceptive activities of TF

**Fig 3:**
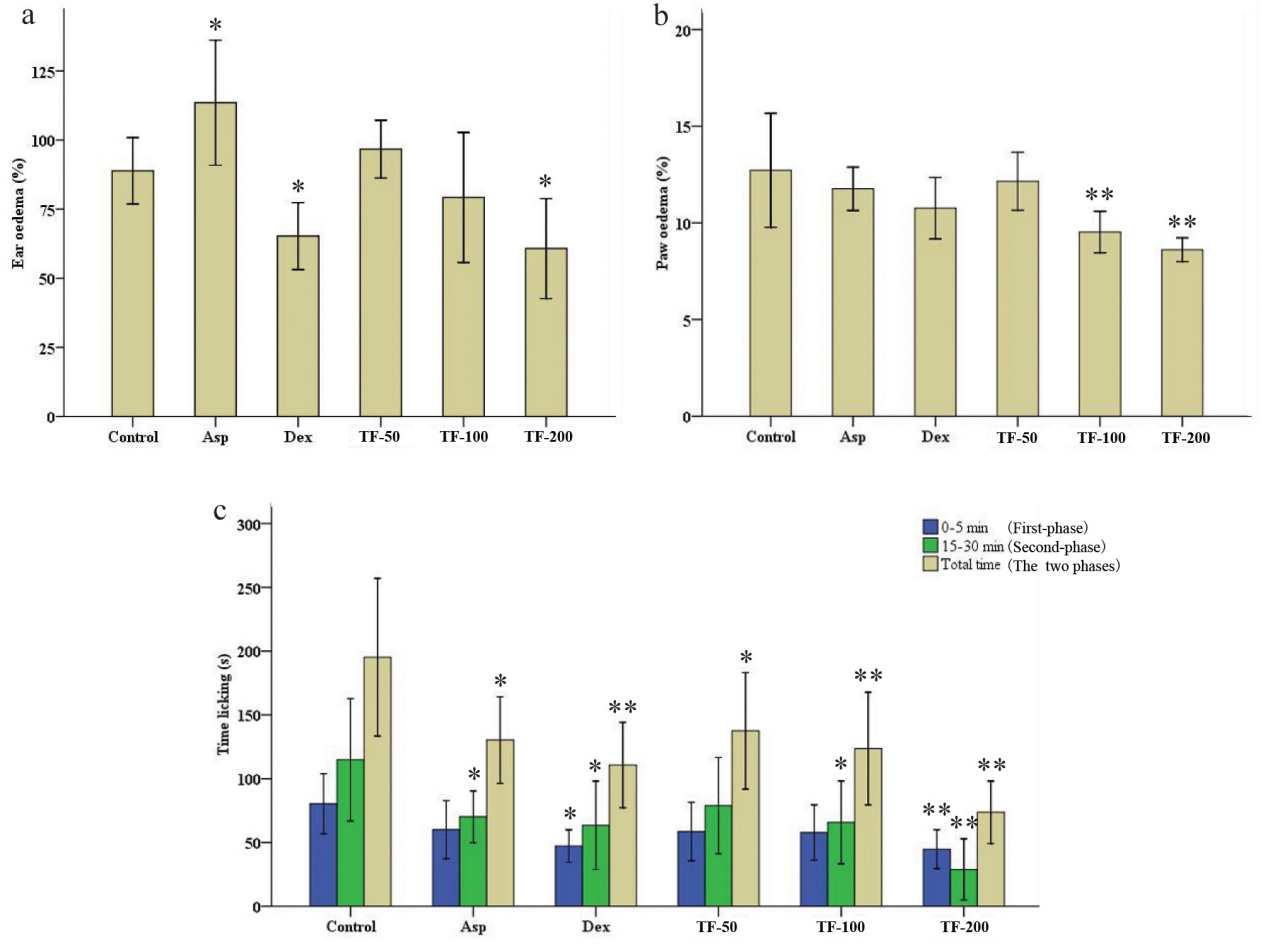
Anti-inflammatory and antinociceptive activities of TF. Groups of KM male mice were pre-treated p.o. with normal saline (control 20 mL/kg), aspirin (Asp 75 mg/20 mL/kg), dexamethasone (Dex 3 mg/20 mL/kg) and total flavonoids (TF) extracts (TF-50: 50 mg/20 mL/kg; TF-100: 100 mg/20 mL/kg; and TF-200: 200 mg/20 mL/kg). One hour after the last drug dose, each group of mice were treated with xylene (a), carrageenan (b) and formalin (c). The licking time was recorded for the first five minutes and minute 15–30 after formalin was injected into the paw. The total time added these two phases. The data were shown as the mean ± SD (n = 10). *p < 0.05 and **p < 0.01 significantly different from the control group.

The xylene-induced ear edema and the carrageenan-induced paw edema were chosen to evaluate the anti-inflammatory activity of TF. Ear edema in the control group was 88.9% (Fig 3a), but was significantly reduced to 65.2% by Dex relative to control. As an analgesic drug, Asp (94.3%, *p* < 0.05) resulted in a higher degree of ear edema on the contrary. TF-200 could significantly inhibit ear edema rate (60.1%, *p* < 0.05) and had a concentration-dependent relationship with TF (50, 100, and 200 mg/20 mL/kg; i.g.). Fig 3b showed paw edema data. The Asp and Dex had a slight reduction in the mice paw edema relative to the control group—12.7% versus 11.8% and 10.8%, respectively. The TF-100 (9.5%, *p* < 0.01) and TF-200 (8.6%, *p* < 0.01) could significantly reduce carrageenan-induced paw edema and had a dose-response relationship with TF concentration (50, 100, and 200 mg/20 mL/kg; i.g.). In general, TF offered dose-dependent inhibition of xylene-induced ear edema and carrageenan-induced paw edema.

The response pattern of the formalin test consists of an initial phase 0–5 min after administration of formalin and a secondary phase 15–30 min after administration. We noted that TF (200 mg/20 mL/kg) caused a significant reduction in the time to paw licking induced by formalin in both phases. The total response times in the TF-treated were 45 s (first-phase, *p* < 0.01), 29 s (second-phase, *p* < 0.01), and 74 s (total time, *p* < 0.01)—values significantly lower than the 80 s, 115 s, and 195 s times seen in control, respectively (Fig 3c). Similar results were also observed in the Dex-treated group, where the pain-response behaviors were inhibited to 47 s (first-phase, *p* < 0.05) and 64 s (second-phase, *p* < 0.05). The Asp also inhibited the pain-response behaviors in the secondary phase. The Asp, Dex, and TF all had antinociceptive activity in formalin test. Meanwhile, the antinociceptive effect of TF was better than those of Asp and Dex. The effect had a dose-dependent relationship with TF (50, 100, and 200 mg/20 mL/kg; i.g.).

### Effects of TF on mediator production in blood of KM male mice

**Fig 4:**
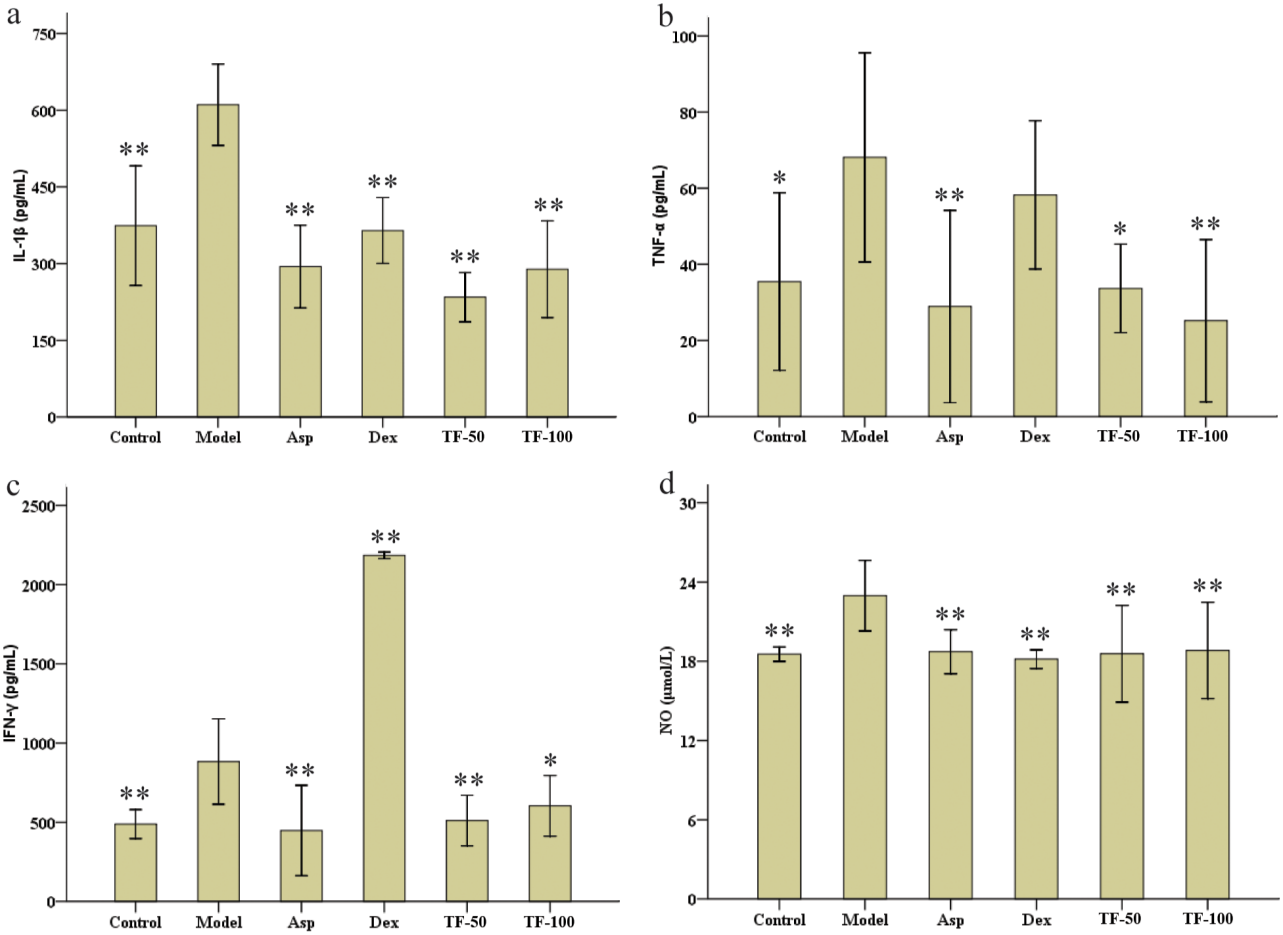
Effects of TF on mediator production in the blood of KM male mice. The KM male mice were pre-treated p.o. with normal saline (control 20 mL/kg), normal saline (model 20 mL/kg), aspirin (Asp 75 mg/20 mL/kg), dexamethasone (Dex 3 mg/20 mL/kg), and total flavonoids (TF) extracts (TF-50: 50 mg/20 mL/kg; and TF-100: 100 mg/20 mL/kg). One hour after the last drug dose, each group except the control group were treated with xylene and formalin to induce inflammation and nociception. The control group was treated with normal saline. The data were shown as mean ± SD (n = 10). *p < 0.05 and **p < 0.01 significantly different from the model group.

We measured four pro-inflammatory cytokines (IL-1β, TNF-α, IFN-γ, and NO) to study the anti-inflammatory and antnociceptive mechanism of TF. As shown in Fig 4a and 4d, relative to the model group (610.6 pg/mL, 23.0 μM), the levels of IL-1β and NO were significantly inhibited by Asp (294.1 pg/mL, 18.7 μM), Dex (364.6 pg/mL, 18.2 μM), TF-50 (234.4 pg/mL, 18.6 μM), and TF-100 (289.1 pg/mL, 18.8 μM). The Asp and TF treatments inhibited pro-inflammatory cytokines TNF-α (Fig 4b) and IFN-γ (Fig 4c) in serum after injury administration. The inhibition of TNF-α showed a dose-dependent relationship with TF (50 and 100 mg/20 mL/kg; i.g.). The Dex reduced TNF-α levels, but not with statistical significance. The increase in IFN-γ (2185.2 pg/mL, *p* < 0.01) was significant (Fig 4b and 4c).

### Effects of TF on NO and cytotoxicity in RAW 264.7 cell

**Fig 5:**
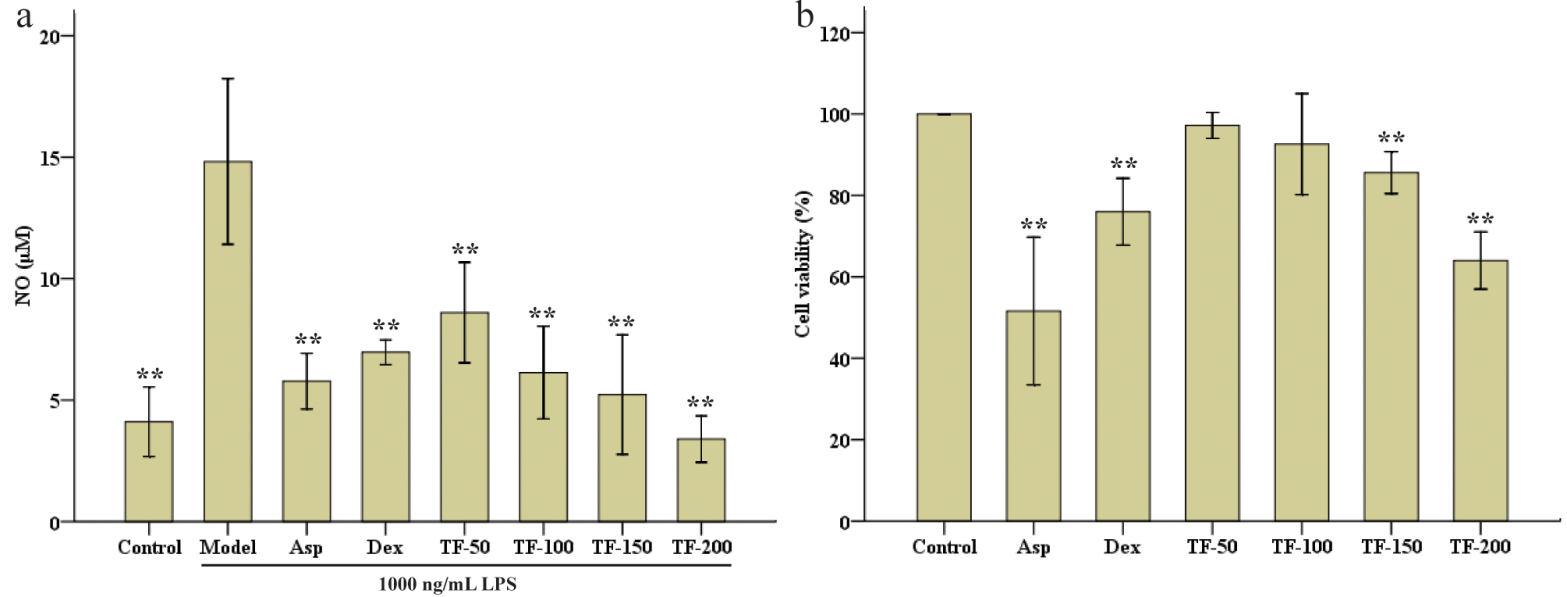
Effects of NO content and the cytotoxicity of TF on RAW 264.7 cells. RAW 264.7 cells were pre-treated with DMEM (control), DMEM (model), and total flavonoids (TF) extracts (TF-50: 50 μg/mL; TF-100: 100 μg/mL; TF-150: 150 μg/mL; and TF-200: 200 μg/mL). Groups except the control group were treated by LPS (100 ng/mL). The control group was treated with DMEM. Cell viability was expressed as a percentage where the control group was set at 100%. Data were shown as mean ± SD (n = 3). *p < 0.05 and **p < 0.01 significantly different from the model group.

As a late inflammatory marker, NO and its associated cytotoxicity were assayed *in vitro*. As shown in Fig 5, LPS increased NO from 4.1 μM (control) to 14.8 μM (model) (Fig 5a). The TF treatments inhibited the production of NO. Indeed, TF-150 and 200 both significantly reduced the levels of NO (*p* < 0.01). The Data in Fig 5b showed that TF has no cytotoxicity when the concentration increased from 50 to 100 μg/mL.

## Discussion

Various studies had shown that flavonoids and polyphenols were the main bioactive components in plants and have antinociceptive and anti-inflammatory properties [12,24,25]. In the present study, we measured TF isolated from black mulberry fruits and found that it was comparable to the levels measured in our previous study (22.2 mg/g) [16]. Dark fruits were rich in TF. The amount of TF in black mulberry (276 mg QE/100 g) was higher than those in white mulberry (29 mg QE/100 g) and red mulberry (219 mg QE/100 g) [5]. The TF levels were also high even compared with other species, such as *Cornus officinalis* and *Hippophae rhamnoides* [26]. Previous studies of flavonoids had emphasized their functions in anti-cancer and protection of liver and lung [27–29]. The flavonoid-rich black mulberry fruits can be as functional foods beneficial to human health.

Five anthocyanins including cyanidin-3-sophoroside, cyanidin-3-glucoside, cyanidin-3-rutinoside, pelargonidin-3-glucoside, and pelargonidin-3-rutinoside (tentative) have been previously identified in mulberry extracts with HPLC-UV [7,15]. In the present study, only two anthocyanins were identified in the black mulberry fruits. The chromatography method we used in this study was effective. The two anthocyanins peaks were well resolved and had a linear relationship that was well supported by quantitative analysis. This different anthocyanins expression were expected because the type and content of the anthocyanins in the fruit were constantly changing during maturation [30,31]. Thus, it is reasonable that the black mulberry fruits contain only two anthocyanins when ripe.

Ascorbic acid (vitamin C; Vc) is a water-soluble vitamin with a five-membered ring polyol. Each hydroxyl can accept a reactive oxygen species. This gives Vc its antioxidant and anti-inflammatory activity [32,33]. We thus compared the antioxidant activities of TF and Vc *in vitro*. TF had more reducing power and scavenging power for OH^-^ and ABTS than Vc, which suggesting that TF has good antioxidant activity and can have potential effects on human health. Vc had stronger scavenging power for ${\text{O}}_{2}^{.-}$ and DPPH than those of TF. As a water-soluble vitamin, Vc may have strong affinity for radicals.

We used xylene to induce inflammation. It is known to cause local increases in capillary permeability, inflammatory cell infiltration, and ear acute exudative inflammatory edema. Acute inflammation induced by xylene is thus frequently used to validate the anti-inflammatory effects of drug in mice, this model is thus quite sensitive to steroidal and non-steroidal anti-inflammatory drugs [22,34]. Carrageenan is a polysaccharide that can cause inflammation, hyperalgesia, and edema in the first phase (0–4 h) of the rising edema and the second phase (4–24 h) in which edema decrease. It has been widely used in animal models to assess anti-edematous activity [11,35]. We found that TF inhibited the first phase. This inhibition was stronger than pear extract [34]. The mechanism may be due to the inhibition of serotonin, bradykinin, and histamine levels [11,36]. Aspirin causes gastrointestinal bleeding and angioedema [37,38], explains in part that the ear edema in aspirin group was bigger than that in control group (Fig 3a). In the formalin test, the response pattern contains two distinct phases. The first phase is caused by direct activation of nociceptors, and the second phase is inflammatory phase release of histamine, serotonin, prostaglandins, and bradykinin [23]. The central and peripheral activities of nociception are clarified by the formalin test [11]. In this study, TF caused a significant reduction in the time to paw licking that was induced by formalin in the two phases. This suggests that TF may reduce the inflammatory pain both centrally and peripherally.

The mechanism of anti-inflammatory and antinociceptive effects are related to many processes including the arachidonic acid metabolic pathway as well as the cytokine, NO, mitogen-activated protein kinase, and nuclear factor κB pathways [9]. The cytokines (IL-1β, IL-6, TNF-α, NO etc.) regulate multiple signaling pathways [39,40]. TF inhibited all four pro-inflammatory cytokines as well as NO in the RAW 264.7 cell line. Another study showed that a plant flavone could also inhibit the production of cyclooxygenase-2 [41]. Therefore, we deduced that TF was a multi-target-directed drug with anti-inflammatory and antinociceptive effects.

We demonstrated that the black mulberry fruits contain rich flavonoids and two kinds of anthocyanins (C3G and C3R). TF may be a multi-target-directed drug with anti-inflammatory and antinociceptive effects. These effects might correlate to its inhibitory activities of pro-inflammatory cytokines. TF is non-cytotoxic at relevant concentrations. These findings suggest that the black mulberry fruits are a valuable source of flavonoids and a natural antioxidant with no toxic side effects. Thus, the fruits are an alternative treatment for inflammatory and nociceptive diseases.
